# Cat abandonment and adoption associated with socioeconomic, veterinary, and trap–neuter–return factors in the Republic of Korea

**DOI:** 10.7717/peerj.21339

**Published:** 2026-06-03

**Authors:** HyungChul Rah

**Affiliations:** Department of Companion Animal and Plant Sciences, Jeonju University, Jeonju, Republic of Korea

**Keywords:** Cat abandonment, Cat adoption, Trap–Neuter–Return (TNR), Socioeconomic determinants, Veterinary service costs

## Abstract

**Background:**

Cat abandonment is a major global animal welfare and public health issue. Millions of cats entering shelters every year and many never reclaimed or adopted. Research on the factors that drive abandonment and adoption in the Republic of Korea is scarce. Cats differ behaviourally and ecologically from dogs, often living as stray or community cats, posing unique management challenges. This study examined how socioeconomic indicators, veterinary costs, and trap–neuter–return (TNR) activities influence cat abandonment and adoption outcomes between 2021 and 2023.

**Methods:**

Publicly available data from 250 Korean jurisdictions, including records of abandonment and adoption, unemployment benefit counts, income indicators, veterinary fees, and TNR activities, were compiled. Ordinary least squares regression identified predictors for two dependent variables: (1) cat abandonments per 100,000 residents and (2) the percentage of abandoned cats adopted. Spatial regression diagnostics assessed the potential spatial dependence.

**Results:**

Across all 3 years, unemployment benefit recipients were negatively associated with cat abandonment rates (β = −0.22 to −0.24), indicating that lower cat abandonments per resident were observed in areas that relied more heavily on unemployment safety nets. In contrast, community cats managed through TNR per 100,000 residents were positively associated with abandonment counts (β = 0.29 to 0.31), likely reflecting reactive TNR targeting in high-abandonment areas or increased detection of abandoned cats. For adoption, rabies vaccination fees were consistently negatively associated (β = −0.19 to −0.25), with revisit consultation fees also showing negative links. In contrast, consultation and hospitalisation fees were positively associated with adoption rates in 2022–2023 (β up to 0.33 for consultation fees), suggesting that better-developed veterinary infrastructure may foster adoption. No spatial dependence was detected for abandonment or adoption, implying that the patterns were shaped by local rather than regional effects.

**Conclusions:**

This study is the first nationwide, multi-year analysis of cat abandonment and adoption in Korea that integrates socioeconomic, veterinary, and TNR data. The findings show that cat abandonments were lower in areas that relied more heavily on unemployment safety nets, while cat abandonments were higher in regions where more community cats were managed through the TNR. Veterinary costs influenced adoption; preventive care costs deterred adopters, while higher consultation and hospitalisation fees reflected stronger veterinary capacity and a culture supportive of adoption. These results broaden our understanding of the drivers of cat welfare outcomes. It further highlights the need for longitudinal and mixed-method studies to clarify causality and guide evidence-based policies to reduce abandonment and improve adoption.

## Introduction

Cat abandonment is a growing global concern, with millions of cats entering shelters each year and substantial numbers never being reclaimed or adopted. Beyond the immediate welfare implications for animals, unmanaged abandonment contributes to public health risks, wildlife predation, and rising municipal costs associated with shelter and control programmes (Kennedy, Cumming & Brown, 2020; Levy, Isaza & Scott, 2014; McDowall et al., 2024; Schmidt et al., 2009).

Historically, cats in the Republic of Korea (ROK) were not primarily regarded as household companion animals but were instead perceived as semi-feral or utilitarian animals, valued mainly for rodent control, and often associated with ambiguity or superstition in traditional society. Until the late twentieth century, dogs were more commonly recognized as companion animals, whereas cats largely existed as free-roaming or community animals (Kim & Chun, 2021; Podberscek, 2009). Since the 2000s, rapid urbanization, declining household size, lifestyle changes, increased exposure to digital media, animal welfare movements, and the expansion of trap–neuter–return (TNR) programs have contributed to a notable shift in public attitudes toward cats (Cho et al., 2020; Kim et al., 2023). Despite this transition, the remnants of earlier perceptions persist and may continue to influence contemporary patterns of cat abandonment, shelter intake, and adoption across regions of Korea. In the ROK, cat abandonment has become increasingly visible in recent years, In the years 2021–2023, over 30% of reported abandonment cases eventually resulted in adoption (Korean Animal Welfare Association (KAWA), 2022, 2023, 2024). Previous empirical studies in the ROK have largely focused on dog abandonment and adoption, reflecting both data availability and the historical emphasis on dogs as companion animals (Rah, 2025; Rah & Choi, 2023; Yoo & Bae, 2022). Although these studies have provided important insights into the socioeconomic and institutional factors associated with dog relinquishment, comparatively little empirical attention has been paid to cats, despite their growing representation in shelters and community settings. In particular, cat abandonment dynamics differ from those of dogs due to species-specific behavioral traits, the prevalence of free-roaming and community cats, and the widespread implementation of TNR programs (Fatjó et al., 2015; Hawes et al., 2020). Cats exhibit various outdoor lifestyles, spanning the stray, feral, free-roaming, and community cat categories, and their survival strategies often make them less reliant on direct human intervention (Horn et al., 2011; Bengsen et al., 2016). These differences create unique challenges for understanding abandonment and adoption trends and highlight the importance of species-specific approaches to welfare policy and shelter management.

Trap–neuter–return (TNR) programs have become a widely used management tool for stray community cats, with the aim of stabilising populations by neutering and returning community cats to their original habitats. The TNR program for community cats was implemented in the ROK in 2018 to control the community cat population. It is carried out by participating veterinary clinics and hospitals with support from the central and local governments of the ROK. While some research shows that targeted, high-coverage TNR can reduce shelter intake and euthanasia rates (Levy, Isaza & Scott, 2014), other studies raise concerns about “reverse causality”, where regions with high abandonment rates may simply conduct more TNR, complicating interpretation of effectiveness (Schmidt et al., 2009; Kilgour et al., 2017). In addition to the TNR, socioeconomic conditions and access to veterinary services play critical roles in shaping the patterns of both abandonment and adoption. Welfare safety nets, such as unemployment benefits, may protect against the abandonment of companion animals during economic hardships (McDowall et al., 2024; Rah, 2025; Weng & Hart, 2012), while veterinary costs can either deter or enable adoption, depending on their accessibility and public subsidies (McDowall et al., 2024; Neal & Kremer, 2024).

Beyond the TNR, socioeconomic conditions and access to veterinary care have been identified as key structural factors shaping patterns of companion animal abandonment and adoption. Financial insecurity can limit the owners’ ability to manage routine or unexpected veterinary expenses. Thereby increasing the likelihood of abandonment during periods of economic stress, whereas welfare support mechanisms may buffer these risks (Rah, 2025; Rah & Choi, 2023; Yoo & Bae, 2022). At the same time, access to veterinary services plays a dual role: high costs may discourage adoption, whereas well-developed veterinary infrastructure can facilitate adoption by supporting animal health and post-adoption care (Hawes et al., 2020). These findings suggest that cat abandonment and adoption are influenced not only by individual decisions but also by broader socioeconomic and service environments. Despite these insights, comprehensive multifactor analyses of cat abandonment and adoption in South Korea are scarce. This study addresses this gap by examining how socioeconomic indicators, TNR activities, and veterinary costs collectively influence cat abandonment and adoption outcomes from 2021 to 2023, thereby offering an evidence base for effective animal welfare policies.

## Materials AND Methods

### Datasets

Public data were collected to investigate the factors contributing to cat abandonment and the adoption of abandoned cats in the ROK as previously described (Rah & Choi, 2023; Rah, 2025). The number of cat abandonments and percentage of cat abandonments adopted were selected as dependent variables (Table S1). Statistics on companion animal abandonment in 250 local cities, counties, and district governments for 2021, 2022, and 2023 were sourced from animal abandonment reports published by the Korean Animal Welfare Association (KAWA) (2022, 2023, 2024). The number of cat abandonments by region, one of the dependent variables, was defined as the number of cats admitted to public animal shelters that were classified as abandoned, excluding cases identified as lost animals that were subsequently returned to their owners. The total number of cats rescued by public animal shelters included abandoned, lost, cats under 3 months of age, and injured cats; however, cats recorded as lost and later returned to their owners were excluded from the abandonment count. The resulting number of cat abandonments was then standardized per 100,000 residents to obtain the number of cat abandonments per 100,000 population. Community cats were excluded from the rescue, whereas cats under 3 months of age and injured cats were included in accordance with the standards of rescue for abandoned animals (The Republic of Korea, 2025). The numbers of cat abandonments by region were from public animal shelters only in the ROK, which are reported to the government and available at the National Animal Protection Information System (https://www.animal.go.kr). This standardisation minimised potential bias due to population size or density, which may co-vary with several socioeconomic indicators, as previously described (Rah, 2025). The percentage of cat abandonments adopted, the second dependent variable, was calculated by dividing the number of adopted abandoned cats by the total number of abandoned cats. To assess the consistency and robustness of the observed associations over time, year-specific regression analyses were conducted using data for 2021, 2022, and 2023. Following our previous study (Rah, 2025), a regression model was first developed using the 2021 data and subsequently applied to the 2022 and 2023 datasets using the exact model specification. This approach allowed if the relationships identified in the baseline year were reproducible in the subsequent years. Thereby reducing the likelihood of year-specific or transient conditions driving the results.

The independent variables included socioeconomic indicators, TNR, and veterinary costs (Table S2). The variables for the socioeconomic indicators by region were the number of people receiving unemployment benefits, comprehensive income tax amounts, and local income. Public data on the number of people receiving unemployment benefits and comprehensive income tax amounts were collected as previously described (Rah & Choi, 2023; Rah, 2025). The regional income data from 2021 to 2023 were collected from the Korean Statistical Information Service (https://kosis.kr). The data from 147 local governments among the 250 local cities, counties, and district governments were used for cat abandonment analysis (Tables 1 and S1). The 2023 Korean map shapefile was obtained from the Geoservice database (http://www.gisdeveloper.co.kr/?p=2332, accessed on 1 June 2025).

**Table 1:** Descriptive statistics of the variables used in the analysis for cat abandonments from 2021 to 2023.

Category	Variables	Observed region	Mean	Standard deviation	Lowest	Highest
Dependent variables	Number of cat abandonments per 100,000 residents in 2021	147	81.20	6.006	2.19	386.41
	Number of cat abandonments per 100,000 residents in 2022	147	79.26	6.059	2.88	366.59
	Number of cat abandonments per 100,000 residents in 2023	147	84.95	6.897	3.39	387.92
	Percentage of cat abandonments adopted in 2021	147	0.33	0.016	0.00	1.00
	Percentage of cat abandonments adopted in 2022	147	0.30	0.014	0.00	1.00
	Percentage of cat abandonments adopted in 2023	147	0.31	0.015	0.05	1.00
Socioeconomic status	Number of people receiving unemployment benefits in 2021	147	11,349.51	673.312	890.00	42,567.00
	Number of people receiving unemployment benefits in 2022	147	10,475.27	619.377	964.00	39,853.00
	Number of people receiving unemployment benefits in 2023	147	10,764.19	650.585	923.00	41,520.00
	Comprehensive income tax amounts (thousand KRW) in 2021	147	284,870,897.96	43,813,451.601	6,000.00	4,599,753,000.00
	Comprehensive income tax amounts (thousand KRW) in 2022	147	311,300,095.24	46,068,685.119	8,113,000.00	4,865,729,000.00
	Comprehensive income tax amounts (thousand KRW) in 2023	147	331,094,000.00	49,089,895.584	9,326,000.00	5,209,045,000.00
	Local income per region (thousand KRW) in 2021	147	123,498,105.82	16,965,747.076	4,136,399.00	1,613,516,055.00
	Local income per region (thousand KRW) in 2022	147	151,856,124.52	21,216,288.130	4,422,652.00	1,996,875,421.00
	Local income per region (thousand KRW) in 2023	147	142,901,479.32	19,049,494.658	4,416,142.00	1,791,445,719.00
Trap–Neuter–Return (TNR)	Number of community cats for TNR per 100,000 residents in 2021	147	191.07	14.835	13.11	1,663.09
	Number of community cats for TNR per 100,000 residents in 2022	147	239.84	20.480	35.89	2,569.73
	Number of community cats for TNR per 100,000 residents in 2023	147	273.47	19.699	58.18	1,839.43
Veterinary costs^1^	Initial examination fee amounts (thousand KRW)	147	9.03	0.115	5.00	15.00
	Revisit examination fee amounts (thousand KRW)	147	7.02	0.121	4.50	11.00
	Consultation fee amounts (thousand KRW)	147	8.26	0.155	3.00	14.00
	Hospitalisation fee amounts for small cats (thousand KRW)	147	50.70	1.000	30.00	100.00
	Rabies vaccination amounts (thousand KRW)	147	24.23	0.312	15.00	30.00
	Feline core vaccination (FHV-1, FCV, FPV) amounts for cats (thousand KRW)	147	39.01	0.346	25.00	45.00
	Complete blood count and interpretation fee amounts (thousand KRW)	147	33.61	0.368	25.00	60.00
	Radiography and interpretation fee amounts (thousand KRW)	147	39.61	0.392	20.00	51.00

**Note:**

1 Reported in 2024 by the Ministry of Agriculture, Food, and Rural Affairs (MAFRA), Republic of Korea and available at https://www.animalclinicfee.or.kr (accessed on 1 June 2025)

TNR activity by region was measured as the number of community cats managed through the TNR, obtained from a survey on the status of companion animal protection and welfare in 2021, 2022, and 2023 by the National Animal Protection Information System of the ROK (https://www.animal.go.kr, accessed on 19 June 2025). The number of community cats managed through the TNR was also standardised per 100,000 residents to yield the number per 100,000 population and minimise potential bias due to population size or density.

The variables on veterinary costs by region included initial examination fees, revisit examination fees; consultation fees, hospitalisation fees, rabies vaccination fees, feline core vaccination (FHV-1, FCV, and FPV) fees, complete blood count and interpretation fees, and radiography and interpretation fees. These data were obtained from 4,159 veterinary clinics and hospitals, surveyed both online and on-site, and reported by the Ministry of Agriculture, Food and Rural Affairs, ROK, in 2024 (https://www.animalclinicfee.or.kr, accessed on 1 June 2025).

### Multiple regression analyses

Least squares multiple regression analyses were conducted to estimate the number of cat abandonments per 100,000 residents and percentage of cat abandonments adopted in 2021 by region as dependent variables, using IBM^®^ SPSS^®^ Software version 29.000 (IBM SPSS Statistics for Windows, Version 29.0; IBM Corp., Armonk, NY, USA). Veterinary cost data, TNR factor, and socioeconomic indicators for 2021 by region were used as the independent variables. The same analyses were conducted to verify and compare the findings using data from 2022 and 2023.

The spatial autocorrelation was performed to investigate the presence of spatial clusters in the number of cat abandonments per 100,000 residents and the percentage of cat abandonments adopted. Spatial regression models were applied using ordinary least squares (OLS) estimation to examine the correlation between the predictors and dependent variables, including the number of cat abandonments and percentage of cat abandonments adopted, using GeoDa software version 1.22 (Anselin, Syabri & Kho, 2009). Before interpretation, model assumptions were evaluated for both dependent variables (number of cat abandonments and percentage of cat abandonments adopted) across all study years (2021–2023). Linearity was assessed using residual–fitted plots, which showed no systematic curvature, thereby supporting the use of linear specifications. The normality of the residuals was examined using the Jarque–Bera test; deviations from normality were observed but were expected given the skewed distribution of abandonment numbers and the bounded nature of adopted percentages. Residual diagnostics were also performed by visually inspecting histogram plots of the model residuals to assess approximate normality (Figs. S1 and S2). Homoskedasticity was visually assessed using residual–fitted plots, which revealed more pronounced heteroskedasticity in the abandonment models than in the adoption models (Figs. S3 and S4). Accordingly, all regression models were estimated using heteroskedasticity-robust standard errors (HC1 and HC3) to ensure reliable statistical inference (Tables S3–S8).

### Linear mixed-effects model analysis

To account for the longitudinal structure of the data and repeated measurements within regions over time, linear mixed-effects models were employed using IBM^®^ SPSS^®^ Software. Two dependent variables (the number of cat abandonments per 100,000 residents and the percentage of abandoned cats adopted) were analysed separately. Year (2021–2023) was included as a fixed effect to assess temporal consistency of associations, while region was included as a random effect to account for unobserved heterogeneity and within-region correlation over time. All explanatory variables included in the year-specific regression models were entered as fixed effects in the mixed models. Models were estimated using restricted maximum likelihood (REML). Linear mixed-effects models were selected to provide a unified analytical framework for longitudinal data, reduce the risk of inflated type I error associated with multiple year-specific analyses, and improve interpretability of temporal patterns.

## Results

Both cat abandonment and adoption outcomes showed substantial regional variations across all 3 years (2021–2023). The average percentages of cat abandonment in 2021, 2022, and 2023 are 33.1%, 29.6%, and 30.8%, respectively (Table S1). Socioeconomic indicators, veterinary service costs, and TNR activity likewise exhibited wide dispersion across local jurisdictions, underscoring the heterogeneous conditions under which cat abandonment and adoption occur, and providing context for regression results.

### Regression models for the number of cat abandonments per 100,000 residents

Twelve independent variables, including three socioeconomic status predictors, one TNR predictor, and eight veterinary cost predictors, were used to develop a regression model that accounted for the number of cat abandonments per 100,000 residents in 2021 (Table 2). A multiple regression model with all 12 predictors yielded an adjusted coefficient of determination (R²-adjusted) of 0.185, with a statistically significant *p-*value of <0.001. Based on the enter method, a subsequent multiple regression model with two statistically significant predictors, one socioeconomic status predictor and one TNR predictor, produced a slightly lower R²-adjusted value of 0.174, with a significant *p*-value of <0.001. These predictors were the number of people receiving unemployment benefits, with a standardised coefficient of −0.242, and the number of community cats managed through the TNR per 100,000 residents, with a standardised coefficient of 0.289.

**Table 2:** Multiple regression model for number of cat abandonments per 100,000 residents in 2021.

Method	Category	Independent variables	Coefficient	Standardized coefficient	*P*-value	Tolerance	VIF	Durbin-watson	R^2^-adjusted	F-statistics	Significance level
Enter	Socio-economic status	Number of people receiving unemployment benefits	−0.002	−0.196	0.028*	−2.222	1.387	1.662	0.185	3.765	<0.001***
		Comprehensive income tax amounts (thousand KRW)	−9.920 × 10^−9^	−0.072	0.578	−0.558	3.011				
		Local income per region (thousand KRW)	−4.380 × 10^−8^	−0.124	0.342	−0.953	3.023				
	Trap–Neuter–Return (TNR)	Number of community cats for TNR per 100,000 residents	0.128	0.317	<0.001***	3.964	1.145				
	Veterinary costs	Initial examination fee amounts (thousand KRW)	−1.816	−0.035	0.724	−0.354	1.724				
		Revisit examination fee amounts (thousand KRW)	1.409	0.028	0.763	0.302	1.594				
		Consultation fee amounts (thousand KRW)	2.327	0.06	0.497	0.682	1.383				
		Hospitalisation fee amounts for cats (thousand KRW)	0.424	0.07	0.372	0.896	1.11				
		Rabies vaccination amounts (thousand KRW)	0.783	0.041	0.699	0.387	1.979				
		Feline core vaccination (FHV-1, FCV, FPV) amounts for cats (thousand KRW)	1.808	0.104	0.299	1.043	1.79				
		Complete blood count and interpretation fee amounts (thousand KRW)	−0.627	−0.038	0.630	−0.483	1.132				
		Radiography and interpretation fee amounts (thousand KRW)	−1.729	−0.113	0.160	−1.414	1.141				
Enter	Socio-economic status	Number of people receiving unemployment benefits	−0.002	−0.242	0.003**	−3.064	1.106	1.542	0.174	16.395	<0.001***
	Trap–Neuter–Return (TNR)	Number of community cats for TNR per 100,000 residents	0.117	0.289	<0.001***	3.649	1.106				

**Notes:**

* *p* < 0.05.

** *p* < 0.01.

*** *p* < 0.001.

The same approach was applied using 2022 data points to verify the 2021 findings (Table 3). A multiple regression model with all 12 predictors yielded an R²-adjusted value of 0.168 and was statistically significant in explaining the number of cat abandonments per 100,000 residents in 2022 (*p* < 0.001). This was followed by a regression model, including two statistically significant predictors, which yielded an increased R²-adjusted value of 0.181, with a *p*-value <0.001. These predictors were the same as those identified in 2021: the number of people receiving unemployment benefits and number of community cats managed through TNR per 100,000 residents, with standardised coefficients of −0.243 and 0.294, respectively.

**Table 3:** Multiple regression model for number of cat abandonments per 100,000 residents in 2022.

Method	Category	Independent variables	Coefficient	Standardized coefficient	*P*-value	Tolerance	VIF	Durbin-watson	R^2^-adjusted	F-statistics	Significance level
Enter	Socio- economic status	Number of people receiving unemployment benefits	−0.002	−0.201	0.026*	−2.255	1.395	1.620	0.168	3.462	<0.001***
		Comprehensive income tax amounts (thousand KRW)	−7.440 × 10^−9^	−0.057	0.667	−0.431	3.02				
		Local income per region (thousand KRW)	−3.360 × 10^−8^	−0.117	0.369	−0.901	2.986				
	Trap–Neuter–Return (TNR)	Number of community cats for TNR per 100,000 residents	0.097	0.326	<0.001***	3.883	1.24				
	Veterinary costs	Initial examination fee amounts (thousand KRW)	2.225	0.042	0.671	0.426	1.721				
		Revisit examination fee amounts (thousand KRW)	−2.597	−0.052	0.587	−0.545	1.598				
		Consultation fee amounts (thousand KRW)	−0.312	−0.008	0.929	−0.089	1.387				
		Hospitalisation fee amounts for cats (thousand KRW)	0.187	0.031	0.701	0.385	1.131				
		Rabies vaccination amounts (thousand KRW)	0.314	0.016	0.880	0.152	2				
		Feline core vaccination (FHV-1, FCV, FPV) amounts for cats (thousand KRW)	1.524	0.087	0.392	0.859	1.805				
		Complete blood count and interpretation fee amounts (thousand KRW)	−1.387	−0.084	0.296	−1.049	1.131				
		Radiography and interpretation fee amounts (thousand KRW)	−0.643	−0.042	0.612	−0.509	1.172				
Enter	Socio- economic status	Number of people receiving unemployment benefits	−0.002	−0.243	0.003**	−3.067	1.117	1.521	0.181	17.080	<0.001***
	Trap–Neuter–Return (TNR)	Number of community cats for TNR per 100,000 residents	0.087	0.294	<0.001***	3.718	1.117				

**Notes:**

* *p* < 0.05.

** *p* < 0.01.

*** *p* < 0.001.

The findings on the number of cat abandonments per 100,000 residents in 2021 and 2022 were verified using a multiple regression model with 2023 data and the same entry approach. Twelve independent predictors were used to develop a regression model explaining the number of cat abandonments per 100,000 residents by 2023 (Table 4). A multiple regression model with all 12 predictors yielded an adjusted coefficient of determination of 0.257 and statistical significance in explaining the number of cat abandonments per 100,000 residents by 2023 (*p* < 0.001). The second regression model, which included three statistically significant predictors, presented a slightly lower adjusted coefficient of determination of 0.234, which was also statistically significant (*p* < 0.001). These three predictors included the number of people receiving unemployment benefits, the number of community cats managed through the TNR per 100,000 residents (both of which confirmed the findings from 2021 and 2022), and hospitalisation fees for cats. The standardised coefficients for the three predictors were −0.221, 0.310, and −0.212, respectively.

**Table 4:** Multiple regression model for number of cat abandonments per 100,000 residents in 2023.

Method	Category	Independent variables	Coefficient	Standardized coefficient	*P*-value	Tolerance	VIF	Durbin-watson	R^2^-adjusted	F-statistics	Significance level
Enter	Socio- economic status	Number of people receiving unemployment benefits	−0.002	−0.185	0.034*	−2.142	1.471	1.730	0.257	5.200	<0.001***
		Comprehensive income tax amounts (thousand KRW)	−5.508 × 10^−9^	−0.039	0.751	−0.319	3.009				
		Local income per region (thousand KRW)	−3.218 × 10^−8^	−0.089	0.47	−0.724	2.993				
	Trap–Neuter–Return (TNR)	Number of community cats for TNR per 100,000 residents	0.114	0.328	<0.001***	3.940	1.358				
	Veterinary costs	Initial examination fee amounts (thousand KRW)	10.249	0.172	0.069	1.834	1.725				
		Revisit examination fee amounts (thousand KRW)	−10.292	−0.182	0.045*	−2.023	1.591				
		Consultation fee amounts (thousand KRW)	−5.404	−0.122	0.149	−1.452	1.383				
		Hospitalisation fee amounts for cats (thousand KRW)	−1.614	−0.235	0.002**	−3.104	1.128				
		Rabies vaccination amounts (thousand KRW)	1.731	0.079	0.438	0.778	2.014				
		Feline core vaccination (FHV-1, FCV, FPV) amounts for cats (thousand KRW)	0.103	0.005	0.957	0.055	1.796				
		Complete blood count and interpretation fee amounts (thousand KRW)	1.794	0.096	0.207	1.268	1.132				
		Radiography and interpretation fee amounts (thousand KRW)	0.181	0.01	0.894	0.134	1.17				
Enter	Socio- economic status	Number of people receiving unemployment benefits	−0.002	−0.221	0.007**	−2.729	1.241	1.689	0.234	15.845	<0.001***
	Trap–Neuter–Return (TNR)	Number of community cats for TNR per 100,000 residents	0.108	0.310	<0.001***	3.836	1.239				
	Veterinary costs	Hospitalisation fee amounts for cats (thousand KRW)	−1.451	−0.212	0.005**	−2.854	1.041				

**Notes:**

* *p* < 0.05.

** *p* < 0.01.

*** *p* < 0.001.

### Regression models for the percentage of cat abandonments adopted

Twelve predictors were used to develop a regression model that accounted for the percentage of cat abandonments adopted in 2021 (Table 5). A multiple regression model with all 12 predictors yielded an adjusted coefficient of determination of 0.031, which was not significant (*p* = 0.179). A subsequent model, which included the rabies vaccination amount as a veterinary cost predictor, produced an adjusted coefficient of determination of 0.030 and was statistically significant (*p* = 0.019) in explaining the percentage of abandoned cats adopted in 2021. The amount of rabies vaccination was a statistically significant predictor, with a standardised coefficient of −0.193.

**Table 5:** Multiple regression model for percentage of cat abandonments adopted in 2021.

Method	Category	Independent variables	Coefficient	Standardized coefficient	*P*-value	Tolerance	VIF	Durbin-watson	R^2^-adjusted	F-statistics	Significance level
Enter	Socio- economic status	Number of people receiving unemployment benefits	1.970 × 10^−6^	0.082	0.397	0.85	1.387	2.283	0.031	1.388	0.179
		Comprehensive income tax amounts (thousand KRW)	2.474 × 10^−11^	0.067	0.638	0.471	3.011				
		Local income per region (thousand KRW)	−7.474 × 10^−11^	−0.078	0.583	−0.55	3.023				
	Trap–Neuter–Return (TNR)	Number of stray cats for TNR per 100,000 residents	<0.001	0.154	0.080	1.762	1.145				
	Veterinary costs	Initial examination fee amounts (thousand KRW)	−0.005	−0.032	0.763	−0.303	1.724				
		Revisit examination fee amounts (thousand KRW)	−0.021	−0.156	0.132	−1.515	1.594				
		Consultation fee amounts (thousand KRW)	0.019	0.182	0.059	1.901	1.383				
		Hospitalisation fee amounts for cats (thousand KRW)	0.002	0.112	0.193	1.309	1.11				
		Rabies vaccination amounts (thousand KRW)	−0.012	−0.24	0.038*	−2.092	1.979				
		Feline core vaccination (FHV-1, FCV, FPV) amounts for cats (thousand KRW)	0.002	0.05	0.644	0.463	1.79				
		Complete blood count and interpretation fee amounts (thousand KRW)	−0.001	−0.027	0.760	−0.306	1.132				
		Radiography and interpretation fee amounts (thousand KRW)	0.001	0.017	0.849	0.191	1.141				
Enter	Veterinary costs	Rabies vaccination amounts (thousand KRW)	−0.010	−0.193	0.019*	−2.365	1.000	2.033	0.030	5.592	0.019*

**Note:**

* *p* < 0.05.

Twelve independent predictors for 2022 were used to verify the 2021 findings on the percentage of cat abandonments adopted (Table 6). A multiple regression model that included all 12 predictors yielded an adjusted coefficient of determination of 0.130 (*p* = 0.002), indicating statistical significance. A subsequent model, including four statistically significant predictors, produced an adjusted coefficient of determination of 0.137, which remained significant (*p* < 0.001). These four predictors included the rabies vaccination amount, confirming the 2021 findings, as well as revisit examinations, consultations, and hospitalisation fees for cats. The standardised coefficients for these four predictors were −0.210, −0.196, 0.332, and 0.218, respectively.

**Table 6:** Multiple regression model for percentage of cat abandonments adopted in 2022.

Method	Category	Independent variables	Coefficient	Standardized coefficient	*P*-value	Tolerance	VIF	Durbin-watson	R^2^-adjusted	F-statistics	Significance level
Enter	Socio- economic status	Number of people receiving unemployment benefits	4.570 × 10^−6^	0.197	0.032*	2.161	1.395	2.093	0.130	2.824	0.002**
		Comprehensive income tax amounts (thousand KRW)	−1.468 × 10^−11^	−0.047	0.726	−0.351	3.02				
		Local income per region (thousand KRW)	9.931 × 10^−12^	0.015	0.913	0.11	2.986				
	Trap–Neuter–Return (TNR)	Number of stray cats for TNR per 100,000 residents	7.125 × 10^−5^	0.102	0.240	1.181	1.24				
	Veterinary costs	Initial examination fee amounts (thousand KRW)	0.001	0.007	0.942	0.072	1.721				
		Revisit examination fee amounts (thousand KRW)	−0.024	−0.199	0.043*	−2.043	1.598				
		Consultation fee amounts (thousand KRW)	0.033	0.359	<0.001***	3.947	1.387				
		Hospitalisation fee amounts for cats (thousand KRW)	0.004	0.256	0.002**	3.117	1.131				
		Rabies vaccination amounts (thousand KRW)	−0.01	−0.214	0.052	−1.958	2				
		Feline core vaccination (FHV-1, FCV, FPV) amounts for cats (thousand KRW)	−0.001	−0.028	0.789	−0.268	1.805				
		Complete blood count and interpretation fee amounts (thousand KRW)	0.002	0.049	0.551	0.597	1.131				
		Radiography and interpretation fee amounts (thousand KRW)	−0.005	−0.132	0.116	−1.582	1.172				
Enter	Veterinary costs	Revisit examination fee amounts (thousand KRW)	−0.023	−0.196	0.021*	−2.343	1.188	2.013	0.137	6.806	<0.001***
		Consultation fee amounts (thousand KRW)	0.031	0.332	<0.001***	3.916	1.217				
		Hospitalisation fee amounts for cats (thousand KRW)	0.003	0.218	0.006**	2.818	1.013				
		Rabies vaccination amounts (thousand KRW)	−0.01	−0.21	0.010*	−2.596	1.108				

**Notes:**

* *p* < 0.05.

** *p* < 0.01.

*** *p* < 0.001.

The findings on the percentage of cat abandonments adopted in 2021 and 2022 were verified using a multiple regression model with the 2023 data and the same entry approach. Twelve independent predictors were used to develop a regression model explaining the percentage of cat abandonments adopted in 2023 (Table 7). A multiple regression model with all 12 predictors yielded an adjusted coefficient of determination of 0.163, with a statistically significant *p*-value < 0.001. A subsequent regression model, including four statistically significant predictors, produced an increased adjusted coefficient of determination of 0.181, with a *p*-value < 0.001. These four predictors include rabies vaccination amounts, (confirming the findings from 2021 and 2022), revisit examination fees, consultation fees and hospitalisation fees for cats, confirming the findings from 2022. The standardised coefficients for these four predictors were −0.250, −0.223, 0.179, and 0.310, respectively.

**Table 7:** Multiple regression model for percentage of cat abandonments adopted in 2023.

Method	Category	Independent variables	Coefficient	Standardized coefficient	*P*-value	Tolerance	VIF	Durbin-watson	R^2^-adjusted	F-statistics	Significance level
Enter	Socio- economic status	Number of people receiving unemployment benefits	2.456 × 10^−6^	0.104	0.258	1.136	1.471	2.146	0.163	3.366	<0.001***
		Comprehensive income tax amounts (thousand KRW)	1.498 × 10^−11^	0.048	0.715	0.366	3.009				
		Local income per region (thousand KRW)	−1.595 × 10^−11^	−0.02	0.880	−0.151	2.993				
	Trap–Neuter–Return (TNR)	Number of stray cats for TNR per 100,000 residents	7.125 × 10^−5^	0.102	0.240	1.181	1.24				
	Veterinary costs	Initial examination fee amounts (thousand KRW)	5.531 × 10^−5^	0.071	0.422	0.806	1.358				
		Revisit examination fee amounts (thousand KRW)	−0.012	−0.088	0.379	−0.883	1.725				
		Consultation fee amounts (thousand KRW)	−0.026	−0.202	0.036*	−2.117	1.591				
		Hospitalisation fee amounts for cats (thousand KRW)	0.019	0.192	0.033*	2.156	1.383				
		Rabies vaccination amounts (thousand KRW)	0.005	0.296	<0.001***	3.683	1.128				
		Feline core vaccination (FHV-1, FCV, FPV) amounts for cats (thousand KRW)	−0.013	−0.268	0.014*	−2.498	2.014				
		Complete blood count and interpretation fee amounts (thousand KRW)	0.002	0.035	0.734	0.341	1.796				
		Radiography and interpretation fee amounts (thousand KRW)	2.542 × 10^−5^	0.126	0.121	1.561	1.132				
Enter	Veterinary costs	Revisit examination fee amounts (thousand KRW)	−0.028	−0.223	0.007**	−2.732	1.188	2.105	0.181	9.090	<0.001***
		Consultation fee amounts (thousand KRW)	0.018	0.179	0.032*	2.172	1.217				
		Hospitalisation fee amounts for cats (thousand KRW)	0.005	0.311	<0.001***	4.128	1.013				
		Rabies vaccination amounts (thousand KRW)	−0.012	−0.25	0.002**	−3.177	1.108				

**Notes:**

* *p* < 0.05.

** *p* < 0.01.

*** *p* < 0.001.

### Spatial regression models for the number of cat abandonments per 100,000 residents and percentage of cat abandonments adopted

Geospatial autocorrelation and clustering of the dependent variables (the number of cat abandonments per 100,000 residents and the percentage of cat abandonments adopted) were analysed using OLS estimation and spatial regression models. Across all the study years (2021–2023), the residual–fitted plots indicated that the linearity assumption was satisfied for both the number of cat abandonments per 100,000 residents and the percentage of cat abandonments adopted, with residuals centred around zero and no systematic curvature observed. The deviations from normality in the residuals were detected using the Jarque–Bera test, which was consistent with the skewed distribution of abandonment rates and the bounded nature of the adoption rates (Tables 8 and 9). The residual variance patterns differed by outcome; abandonment models showed increasing variance at higher fitted values, whereas adoption models exhibited relatively stable variance across fitted values (Figs. S1 and S2). When heteroskedasticity-robust standard errors (HC1 and HC3) were applied, the direction and statistical significance of the key coefficients remained consistent across years, indicating that the main results were robust to violations of classical OLS assumptions (Tables S3–S8).

**Table 8:** Results of ordinary least squares models assessing number of cat abandonments per 100,000 residents and spatial dependence from 2021 to 2023.

Variable		2021		2022		2023	
		coefficient	*p*-value	Coefficient	*p*-value	Coefficient	*p*-value
Constant		0.345	0.943	0.541	0.913	0.503	0.925
*Socio-economic status*	Number of people receiving unemployment benefits	−0.002	0.004**	−0.002	0.003**	−0.002	0.005**
*Trap–Neuter–Return (TNR)*	Number of community cats for TNR per 100,000 residents	0.130	<0.001***	0.100	<0.001***	0.118	<0.001***
*Veterinary costs*	Initial examination fee amounts (thousand KRW)	−1.801	0.639	3.283	0.402	10.068	0.017*
	Revisit examination fee amounts (thousand KRW)	1.967	0.579	−3.378	0.351	−10.038	0.009**
	Hospitalisation fee amounts for cats (thousand KRW)	0.477	0.174	0.200	0.576	−1.536	<0.001***
	Complete blood count and interpretation fee amounts (thousand KRW)	−0.355	0.700	−0.603	0.520	2.333	0.022*
Lambda (λ)		–	–	–	–	–	–
R^2^		0.505	–	0.484	–	0.518	–
Log likelihood		−1,323.520	–	−1,328.300	–	−1,346.090	–
Akaike info criterion		2,673.040	–	2,682.590	–	2,718.170	–
Schwarz criterion		2,718.820	–	2,728.370	–	2,763.950	–
Likelihood ratio test		–	–	–	–	–	–
Jarque-Bera test		221.503	<0.001***	479.070	<0.001**	202.189	<0.001***
Breusch-Pagan test		167.835	<0.001***	151.950	<0.001***	133.282	<0.001***
Moran’s I (error)		0.057	0.276	0.062	0.239	−0.006	0.996
Lagrange multiplier (lag)		1.897	0.168	2.119	0.145	0.166	0.684
Robust LM (lag)		0.995	0.319	1.044	0.307	0.657	0.418
Lagrange multiplier (error)		0.934	0.334	1.110	0.292	0.012	0.914
Robust LM (error)		0.031	0.859	0.035	0.852	0.503	0.478

**Notes:**

* *p* < 0.05.

** *p* < 0.01.

*** *p* < 0.001.

**Table 9:** Results of ordinary least squares models assessing the percentage of cat abandonments adopted and spatial dependence from 2021 to 2023.

Variable		2021		2022		2023	
		Coefficient	*p*-value	Coefficient	*p*-value	Coefficient	*p*-value
Constant		0.003	0.840	0.002	0.856	0.002	0.905
*Trap–Neuter–Return (TNR)*	Number of community cats for TNR per 100,000 residents	<0.001	0.014*	<0.001	0.047*	<0.001	0.165
*Veterinary costs*	Revisit examination fee amounts (thousand KRW)	−0.016	0.142	−0.021	0.022*	−0.023	0.012*
	Consultation fee amounts (thousand KRW)	0.021	0.007**	0.034	<0.001***	0.020	0.003**
	Hospitalisation fee amounts for cats (thousand KRW)	0.002	0.028*	0.004	<0.001***	0.005	<0.001***
	Rabies vaccination amounts (thousand KRW)	−0.013	0.004**	−0.011	0.006**	−0.014	<0.001***
	Core vaccination (FHV-1, FCV, FPV) amounts for cats (thousand KRW)	0.007	0.042*	0.002	0.405	0.004	0.197
	Complete blood count and interpretation fee amounts (thousand KRW)	0.001	0.623	0.004	0.074	0.007	0.006**
Lambda (λ)		–	–	–	–	–	–
R^2^		0.577	–	0.626	–	0.636	–
Log likelihood		129.132	–	173.645	–	164.760	–
Akaike info criterion		−232.264	–	−321.290	–	−303.521	–
Schwarz criterion		−186.485	–	−275.511	–	−257.742	–
Likelihood ratio test		–	–	–	–	–	–
Jarque-Bera test		143.285	<0.001***	166.496	<0.001***	169.843	<0.001***
Breusch-Pagan test		153.264	<0.001***	241.570	<0.001***	188.072	<0.001***
Moran’s I (error)		0.025	0.594	−0.026	0.745	0.010	0.776
Lagrange multiplier (lag)		0.155	0.694	0.001	0.976	0.013	0.909
Robust LM (lag)		1.045	0.307	0.224	0.636	<0.001	0.991
Lagrange multiplier (error)		0.172	0.679	0.191	0.662	0.028	0.866
Robust LM (error)		1.062	0.303	0.415	0.520	0.015	0.902

**Notes:**

* *p* < 0.05.

** *p* < 0.01.

*** *p* < 0.001.

For the number of cat abandonments per 100,000 residents, none of the diagnostic tests for spatial dependence were statistically significant, indicating the absence of spatial dependence in 2021, 2022, and 2023 (Table 8 and Fig. S5). The likelihood ratio tests for both spatial models were not significant (*p* > 0.05), suggesting that neither model outperformed the OLS model in explaining the number of cat abandonments per 100,000 residents. Among the 12 predictors, the number of people receiving unemployment benefits (a socioeconomic indicator) was negatively associated with the number of cat abandonments, whereas the number of community cats managed through the TNR per 100,000 residents (a TNR predictor) was consistently positively associated (both with statistical significance) across all 3 years. These two predictors were significant in the regression models for annual cat abandonments per 100,000 residents.

None of the diagnostic tests for spatial dependence were significant for the percentage of cat abandonments adopted, indicating the absence of spatial dependence from 2021 to 2023 (Table 9 and Fig. S6). The likelihood ratio test for any of the spatial models was also not significant (*p* > 0.05), suggesting that neither spatial model outperformed OLS. The three veterinary cost predictors were statistically significant across all 3 years. The consultation and hospitalisation fees for cats were positively associated with the percentage of cat abandonments adopted, whereas rabies vaccination amounts were negatively associated. In the regression models, rabies vaccination amounts were significant for all 3 years; and consultation and hospitalisation fees for cats were significant in 2022 and 2023.

### Linear mixed-effects model results

For the number of cat abandonments per 100,000 residents, the linear mixed-effects model identified a few significant predictors (Table S9). The number of community cats for TNR per 100,000 residents was significantly associated with cat abandonment, whereas the number of people receiving unemployment benefits was negatively associated with cat abandonment. These associations were consistent with those observed in the year-specific regression analyses, indicating robustness of the findings across different analytical approaches. The effect of year was statistically significant, indicating a systematic change over time in cat abandonments after accounting for covariates.

For the percentage of abandoned cats adopted, the mixed-effects model also revealed several significant associations (Table S10). The consultation and hospitalisation fees for cats were positively associated with the percentage of cat abandonments adopted, whereas rabies vaccination and revisit examination fees were negatively associated with that percentage. The direction and magnitude of these associations were consistent with those obtained from the year-specific regression models. The fixed effect of year was not significant, indicating that cat adoption rates were relatively stable across years.

## Discussion

This study aimed to identify the factors contributing to the cat abandonment and adoption of abandoned cats in the ROK by addressing three questions, following a prior report on dog abandonment (Rah, 2025). Accordingly, this study addresses the following questions at the regional level: (1) How are socioeconomic status, veterinary cost, and TNR activity associated with regional variation in cat abandonment in the ROK during the study period from 2021 to 2023? (2) How are these same factors related to regional differences in the adoption of abandoned cats across local jurisdictions in the ROK? (3) Is there any evidence of spatial dependence in cat abandonment or adoption in the ROK?

A key finding addressing the first question was that the number of people receiving unemployment benefits (a socioeconomic indicator) and the number of community cats managed through the TNR per 100,000 residents (a TNR indicator) were consistently associated with the number of cat abandonments per 100,000 residents in all 3 years. This suggests that regions with more unemployment benefit recipients may experience fewer cat abandonments per resident, when standardised by population size. From a socioeconomic standpoint, this may suggest that cat abandonments per resident were lower in regions that relied more heavily on unemployment benefits. The study also found that regions with more community cats undergoing TNR exhibited high abandonment rates. This may reflect either a greater underlying level of abandonment, prompting TNR responses, or increased detection and documentation of abandoned cats because of TNR activity. This finding highlights the need for longitudinal research to clarify causal relationships.

The findings on the number of cat abandonments per 100,000 residents are consistent with those of prior research on dog abandonment in Korea, which also reported a negative association between the number of recipients of unemployment benefits and the number of dog abandonments per 100,000 residents (Rah, 2025). However, this study extends the boundary of the evidence by confirming that the same relationship holds true for cat abandonment. Notably, while an earlier study on dogs identified significant spatial dependence in abandonment patterns, the current spatial diagnostics showed no clustering for cats over the 3 years. This contrast implies that cat abandonment may be driven less by regional spillover effects and more by localised socioeconomic conditions. Thus, these findings not only validate but also refine previous conclusions by demonstrating that unemployment-related welfare access may reduce the abandonment of both dogs and cats. Meanwhile, also pointing to key differences in the geographic dynamics of dog and cat abandonment. This emphasises the importance of tailoring animal welfare policies to species-specific behaviour and spatial patterns.

Another key finding addressing the second research question was that rabies vaccination amounts, used as a proxy for veterinary service accessibility, were consistently and negatively associated with the percentage of abandoned cats adopted across all 3 years. Additionally, higher revisit examination fees are negatively associated with adoption rates, whereas higher consultation and hospitalisation fees are positively associated with adoption rates in 2022 and 2023. Although these associations appear counterintuitive, they may involve two distinct mechanisms. Higher revisit examination fees likely reflect the reduced affordability of follow-up care, which has been identified as a barrier to companion animal ownership and veterinary service use, particularly in economically disadvantaged households (Kitole, 2025; Rah, 2025; Yan & Teng, 2023). In contrast, higher consultation and hospitalisation fees may indicate regions with more developed veterinary infrastructure and higher overall socioeconomic resources, where potential adopters have greater capacity to provide ongoing care, thereby supporting higher adoption rates (Bradley & Rajendran, 2021; Rah, 2025; Yan & Teng, 2023). Collectively, these patterns suggest that lower adoption rates are linked to limited access to affordable follow-up care. In contrast, higher adoption rates may be characteristic of regions with better-resourced veterinary services and greater community investment in companion animals.

The findings on the number of cat abandonments per 100,000 residents align partially with those of a U.S. study, which demonstrated that high-impact, targeted TNR combined with adoption significantly reduced shelter intake in Florida. However, their work emphasised the TNR’s proactive role, whereas the current results raise the possibility of reverse causality, where the TNR follows abandonment spikes rather than preventing them (Levy, Isaza & Scott, 2014). A U.S. study further noted that short-term TNR efforts in New York City did not produce immediate population reductions due to migration and turnover, supporting our interpretation that TNR counts may reflect ongoing abandonment pressures rather than solely mitigating them (Kilgour et al., 2017). Similarly, another U.S. study modelled TNR and euthanasia strategies and highlighted that immigration and movement between unmanaged populations can undermine the effectiveness of TNR unless sterilisation coverage is extremely high (Schmidt et al., 2009). This concern was reinforced by an Israeli report that conducted a 12-year field study and found that only high-intensity, spatially contiguous neutering efforts led to sustainable reductions in free-roaming cat populations (Gunther et al., 2022). Together, these studies suggest that insufficient TNR intensity and lack of coordinated spatial implementation can produce compensatory effects, such as increased immigration or reproduction in unmanaged areas. The present findings indicate that regional TNR counts in Korea may function less as indicators of preventive success compared to prior studies. Rather, the counts serve as markers of persistent abandonment pressure, particularly when evaluated using aggregate regional data. Unlike studies that assessed carefully targeted or experimentally designed TNR interventions, the current analysis captured routine, administratively reported TNR activities, which may explain why regions with higher TNR levels continue to experience elevated abandonment rates. By explicitly contrasting proactive and reactive interpretations of the TNR, this study extends prior research and underscores the importance of multi-year, large-scale, and spatially coordinated strategies when interpreting TNR metrics in animal welfare policy.

The findings on the adopted percentage of cat abandonments built on work by an Australian group emphasised that structural barriers to veterinary access can negatively influence owner engagement with preventive care and long-term pet retention (McDowall et al., 2024). The findings of the present study extend this insight by demonstrating that adoption and ownership are linked to local veterinary cost environments. These findings suggest that formal veterinary infrastructure and economic accessibility of veterinary services play an important role in shaping regional adoption outcomes. The regions with more developed veterinary services may provide more favourable conditions for adoption by supporting animal health management and post-adoption care. However, this study did not include measures of informal social networks or community-based support systems. Therefore, the relative importance of formal veterinary infrastructure compared with informal mechanisms could not be directly assessed. Accordingly, the observed associations should be interpreted as reflecting the role of formal service environments, rather than broader social support dynamics.

To further examine the robustness of the findings across time, linear mixed-effects models were applied to account for repeated measurements within regions. This approach allowed the study to assess temporal consistency within a unified modeling framework while reducing the potential inflation of type I error associated with separate yearly analyses. The results showed that year had a significant effect on cat abandonment but not on adoption rates, indicating that abandonment levels varied over time, whereas adoption rates remained relatively stable. Importantly, the key predictors identified in the year-specific regression analyses remained consistent in both direction and statistical significance, supporting the temporal stability and robustness of the observed associations.

One notable finding in addressing the third research question was that none of the diagnostic tests for spatial dependence was statistically significant for either the number of cat abandonments per 100,000 residents or the percentage of cat abandonments adopted, indicating the absence of spatial clustering in these outcomes across the 3 years. Taken together, the absence of spatial dependence across all 3 years suggests that cat abandonment and adoption in the ROK are primarily driven by local socioeconomic and service-related conditions rather than by geographic spillover effects. The consistent role of unemployment-related indicators suggests that cat abandonments per resident were lower in areas that relied heavily on unemployed safety nets. In contrast, the positive association between TNR activity and abandonment rates likely reflects a reactive dynamic, whereby municipalities with higher levels of abandonment intensify their population management efforts rather than directly increasing abandonment. With respect to adoption, the observed associations with veterinary cost indicators highlight the importance of access to veterinary infrastructure and affordability of care. Regions characterised by limited access to affordable preventive or follow-up services may face greater barriers to adoption, whereas areas with more developed veterinary systems may provide conditions more conducive to successful adoption. Overall, these findings highlight that structural economic conditions and veterinary service accessibility play a more central role than spatial proximity alone in shaping cat abandonment and adoption outcomes.

The findings on the lack of spatial dependence for either the number of cat abandonments per 100,000 residents or the percentage of cat abandonments adopted differ from previous reports. These include a Canadian study on the geospatial analysis of animals surrendered to shelters (dogs and cats), and a Korean study on dog abandonment and adoption (Ly, Gordon & Protopopova, 2021). The Canadian study identified differences in the deprivation and animal relinquishment risks in various geographic areas (Ly, Gordon & Protopopova, 2021). The Korean study with dog-focused research showed that dog abandonment tends to cluster in specific areas, whereas spatial patterns in adoption are less consistent, suggesting that dogs may be influenced by the dynamics of neighbouring regions (Rah, 2025). The current results extend previous findings by showing that, for cats, socioeconomic and veterinary infrastructure factors, not spatial proximity, better explain abandonment and adoption patterns. Studies on cat movement ecology provide context for this finding, having demonstrated that feral and free-roaming cats exhibit highly variable home ranges and activity patterns depending on local resource availability (Bengsen et al., 2016; Horn et al., 2011). This suggests that abandonment and TNR responses may manifest differently across regions, even in the absence of spatial autocorrelation. Similarly, a New Zealand study found that feral cats adjust their space use dynamically to optimise access to key resources and tolerate conspecifics in high-value areas, implying that TNR counts may reflect the underlying aggregation in resource-rich or high-abandonment zones (Recio & Seddon, 2013). Through the integrating of these ecological perspectives with socioeconomic and veterinary cost data, this study adds value by demonstrating that cat abandonment and adoption dynamics are shaped by the interplay of economic safety nets, veterinary service accessibility, and behavioural ecology. Furthermore, simplistic assumptions about geographic clustering or unidirectional TNR effects are insufficient for designing effective policy interventions.

These findings should also be interpreted in light of the historically ambivalent position of cats in Korean society, where they have long occupied the space between companion animals and free-roaming community animals. Such perceptions partly explain why structural factors such as socioeconomic conditions and veterinary infrastructure exert a more substantial influence on abandonment and adoption patterns than spatial proximity alone. Amid recent shifts toward greater acceptance of cats as companions, lingering cultural norms may continue to shape regional differences in how cats are abandoned, managed, and adopted. This study had several limitations. It draws on open public datasets, such as the Korean Animal Welfare Association reports, Ministry of Agriculture, Food and Rural Affairs veterinary cost data, and TNR counts, rather than data collected directly in the field. While public datasets offer national coverage and longitudinal consistency, enabling a multiyear, nationwide analysis that would be impractical for a single research group, they may also introduce inconsistencies in data collection methods, underreporting, or classification errors, such as misidentifying abandoned cats as lost (Delavenne et al., 2025; Grace et al., 2024; McCluskey, 2021; O’Neill et al., 2014). Furthermore, one of the key findings, that the positive association between the number of community cats in TNR programs and number of cat abandonments may reflect reverse causality, highlights a known limitation in TNR research (Coe et al., 2021; Gunther et al., 2022; Lepczyk, Longcore & Rich, 2022). High-abandonment regions may conduct more TNR programmes, rather than the programs causing more abandonment. Reverse causality is a well-documented issue in this field, and TNR metrics must therefore be interpreted cautiously in abandonment studies (Schmidt et al., 2009; Levy, Isaza & Scott, 2014). The present study underscores the need for multi-year longitudinal tracking to verify causal pathways. Although the models in this study identified meaningful associations, they explained only a limited proportion of the total variability, reflecting the complexity of cat abandonment and adoption processes. Unmeasured behavioural and contextual factors likely contribute to this unexplained variation, underscoring the need for longitudinal and mixed-methods research. Finally, although this study incorporated socioeconomic indicators, TNR activity, and veterinary costs into the regression models, it did not include human behavioural factors, such as attitudes towards spaying and neutering or public awareness of shelters (Xu & Jiang, 2023; Gunther, Levin & Klement, 2025; Hiby et al., 2025). These unmeasured variables may have influenced the abandonment and adoption outcomes. Future research should adopt mixed-methods designs that combine quantitative models with qualitative insights into cultural and behavioural determinants. These findings highlight that improving cat welfare requires not only population management strategies but also stronger human-welfare services and greater access to affordable veterinary care. Integrating social support policies with animal welfare initiatives may help to reduce cat abandonment and improve adoption outcomes.

## Conclusions

This study identified the socioeconomic, veterinary, and TNR factors associated with cat abandonment and adoption in the ROK from 2021 to 2023. Across the 3 years, the number of unemployment benefit recipients, a proxy for welfare access, was consistently and negatively associated with the number of cat abandonments, suggesting fewer cat abandonments per resident were fewer in areas that relied more heavily on unemployment safety nets. Conversely, the number of community cats managed through TNR programs was positively associated with abandonment. This may reflect either increased TNR activity in high-abandonment regions or improved detection of abandoned cats through TNR efforts, underscoring the need for longitudinal research to disentangle the causal pathways. Adoption outcomes were largely shaped by veterinary cost indicators. High rabies vaccination costs and revisit consultation fees were associated with low adoption rates, whereas high consultation and hospitalisation fees were positively associated with adoption, suggesting that regions with more advanced veterinary infrastructure and stronger animal care cultures foster more favourable adoption environments. This study expands the existing knowledge by integrating socioeconomic, veterinary, and ecological perspectives, demonstrating that cat abandonment and adoption are influenced less by geography and more by structural access to welfare and veterinary care. Future studies should incorporate longitudinal data and mixed-methods approaches to clarify causality and include cultural and behavioural insights into the design of policies aimed at reducing abandonment and increasing the adoption of abandoned cats.

## Supplemental Information

10.7717/peerj.21339/supp-1 Supplemental Information 1 The Numbers of Cat Abandonments and Their Adoption by Region from 2021 to 2023 used in the Analysis.

10.7717/peerj.21339/supp-2 Supplemental Information 2 The Independent Variables Including Socioeconomic Indicators, TNR, and Veterinary costs used in the Analysis.

10.7717/peerj.21339/supp-3 Supplemental Information 3 Comparison of coefficient estimates and standard errors of the model for the number of cat abandonments per 100,000 residents in 2021 across OLS and heteroskedasticity-robust specifications (HC1 and HC3). Notes: Dependent variable is the number of cat abandonments per 100,000 residents in 2021. HC1 and HC3 denote heteroskedasticity-robust standard errors. Coefficient estimates were identical across the models, and only standard errors and p-values differ. Statistical significance was set at *p* < 0.05.

10.7717/peerj.21339/supp-4 Supplemental Information 4 Comparison of coefficient estimates and standard errors of the model for the percentage of cat abandonments adopted in 2021 across OLS and heteroskedasticity-robust specifications (HC1 and HC3). Notes: Dependent variable is the percentage of cat abandonments adopted in 2021. HC1 and HC3 denote heteroskedasticity-robust standard errors. Coefficient estimates were identical across the models, and only standard errors and p-values differ. Statistical significance was set at *p* < 0.05.

10.7717/peerj.21339/supp-5 Supplemental Information 5 Comparison of coefficient estimates and standard errors of the model for the number of cat abandonments per 100,000 residents in 2022 across OLS and heteroskedasticity-robust specifications (HC1 and HC3). Notes: Dependent variable is the number of cat abandonments per 100,000 residents in 2022. HC1 and HC3 denote heteroskedasticity-robust standard errors. Coefficient estimates were identical across the models, and only standard errors and p-values differ. Statistical significance was set at *p* < 0.05.

10.7717/peerj.21339/supp-6 Supplemental Information 6 Comparison of coefficient estimates and standard errors of the model for the percentage of cat abandonments adopted in 2022 across OLS and heteroskedasticity-robust specifications (HC1 and HC3). Notes: Dependent variable is the percentage of cat abandonments adopted in 2022. HC1 and HC3 denote heteroskedasticity-robust standard errors. Coefficient estimates were identical across the models, and only standard errors and p-values differ. Statistical significance was set at *p* < 0.05.

10.7717/peerj.21339/supp-7 Supplemental Information 7 Comparison of coefficient estimates and standard errors of the model for the number of cat abandonments per 100,000 residents in 2023 across OLS and heteroskedasticity-robust specifications (HC1 and HC3). Notes: Dependent variable is the number of cat abandonments per 100,000 residents in 2023. HC1 and HC3 denote heteroskedasticity-robust standard errors. Coefficient estimates were identical across the models, and only standard errors and p-values differ. Statistical significance was set at *p* < 0.05.

10.7717/peerj.21339/supp-8 Supplemental Information 8 Comparison of coefficient estimates and standard errors of the model for the percentage of cat abandonments adopted in 2023 across OLS and heteroskedasticity-robust specifications (HC1 and HC3). Notes: Dependent variable is the percentage of cat abandonments adopted in 2023. HC1 and HC3 denote heteroskedasticity-robust standard errors. Coefficient estimates were identical across the models, and only standard errors and p-values differ. Statistical significance was set at *p* < 0.05.

10.7717/peerj.21339/supp-9 Supplemental Information 9 Linear mixed-effects model for the number of cat abandonments per 100,000 residents (Region = random intercept, Year = fixed effect).

10.7717/peerj.21339/supp-10 Supplemental Information 10 Linear mixed-effects model for the percentage of cat abandonments adopted (Region = random intercept, Year = fixed effect).

10.7717/peerj.21339/supp-11 Supplemental Information 11 Visual assessment of residual normality using histograms of OLS models for the cat abandonment (per 100,000 residents) across 2021 (left), 2022 (center), and 2023 (right), demonstrating non-normality and potential skewness.

10.7717/peerj.21339/supp-12 Supplemental Information 12 Visual assessment of residual normality using histograms of OLS models for the percentage of cat abandonments adopted in 2021 (left), 2022 (center), and 2023 (right), demonstrating non-normality and potential skewness.

10.7717/peerj.21339/supp-13 Supplemental Information 13 Absence of Spatial Dependence of the number of cat abandonments per 100,000 residents in 2021(left), 2022(center), and 2023(right).

10.7717/peerj.21339/supp-14 Supplemental Information 14 Absence of Spatial Dependence of the percentage of cat abandonments adopted in 2021(left), 2022(center), and 2023(right).

10.7717/peerj.21339/supp-15 Supplemental Information 15 Absence of Spatial Dependence of the number of cat abandonments per 100,000 residents in 2021(left), 2022(center), and 2023(right).

10.7717/peerj.21339/supp-16 Supplemental Information 16 Absence of Spatial Dependence of the percentage of cat abandonments adopted in 2021(left), 2022(center), and 2023(right).

## Additional Information and Declarations

### Data Availability

The following information was supplied regarding data availability:

The raw measurements are available in the Supplemental Files.
