# Supplementary figures and images for: Cat abandonment and adoption associated with socioeconomic, veterinary, and trap–neuter–return factors in the Republic of Korea
Source: PeerJ. 2026 Jun 3;14:e21339. doi: 10.7717/peerj.21339 (PMC13242195; doi:10.7717/peerj.21339)

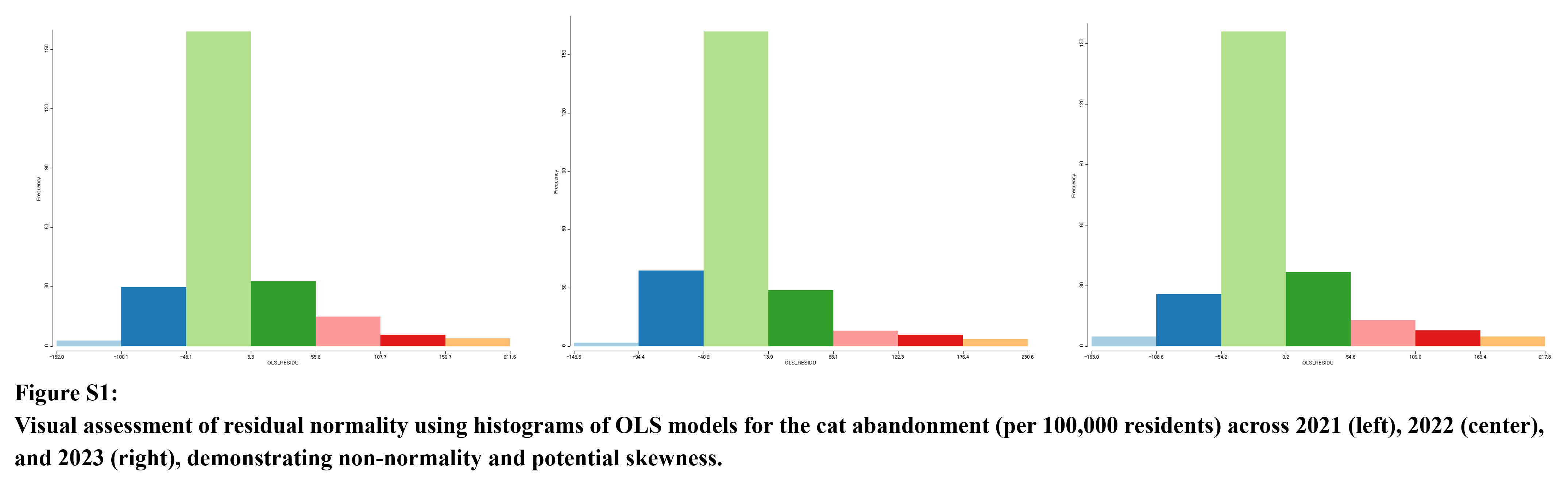

Supplement: Supplemental Information 11 [file peerj-14-21339-s011.png]

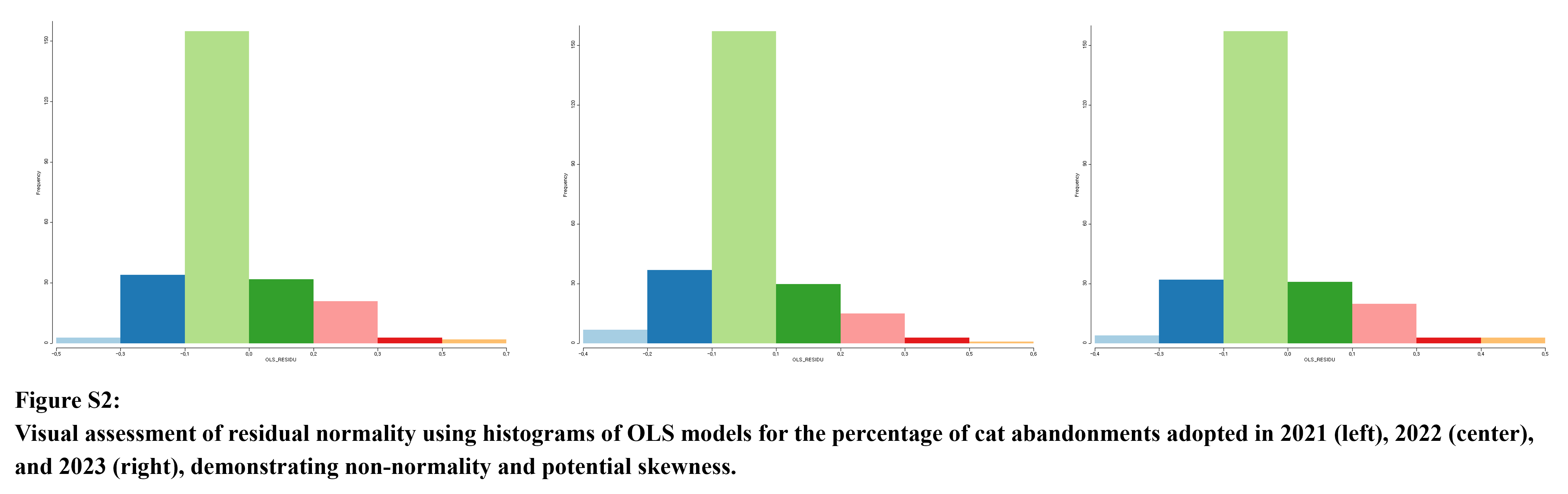

Supplement: Supplemental Information 12 [file peerj-14-21339-s012.png]

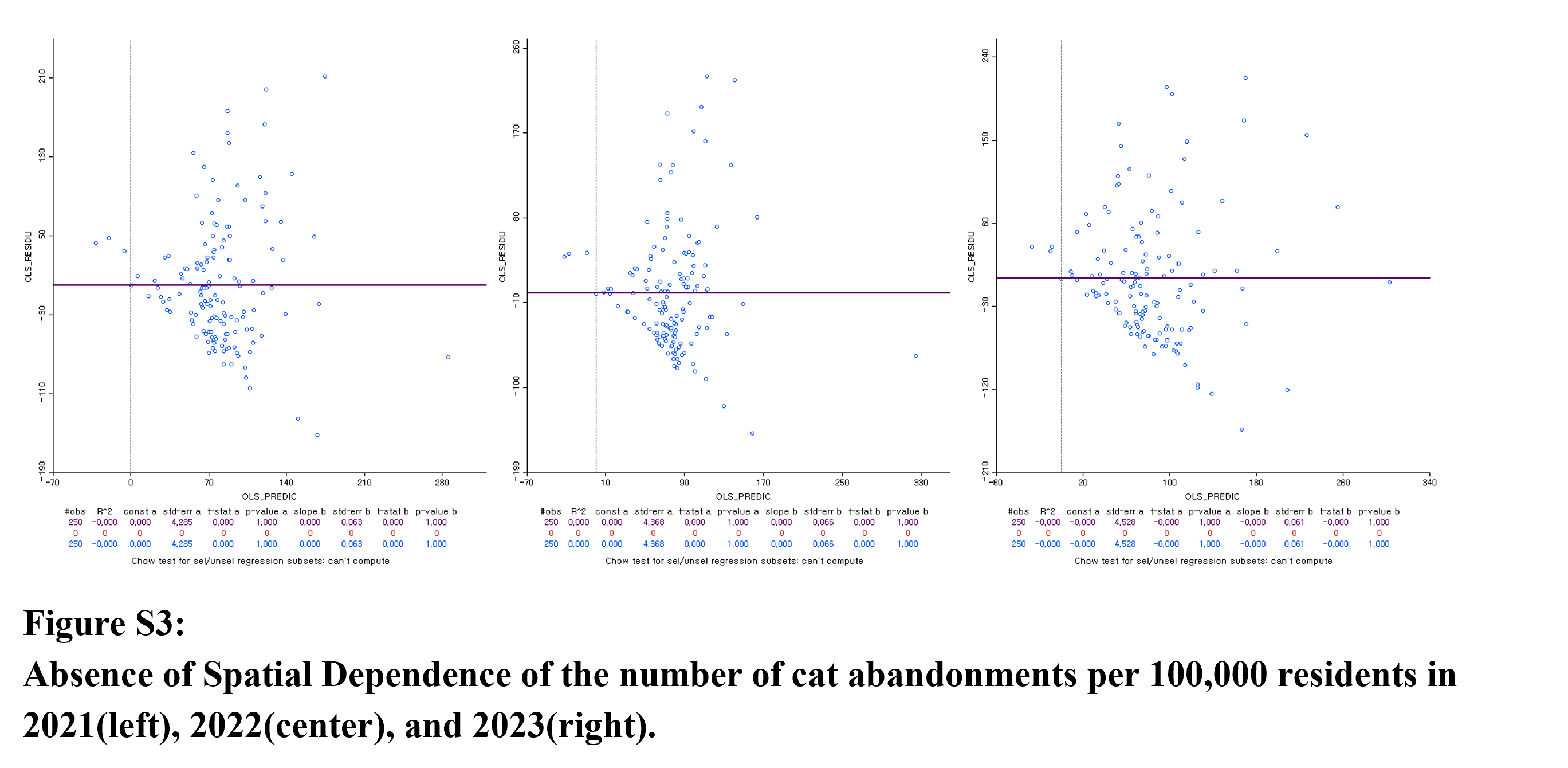

Supplement: Supplemental Information 13 [file peerj-14-21339-s013.png]

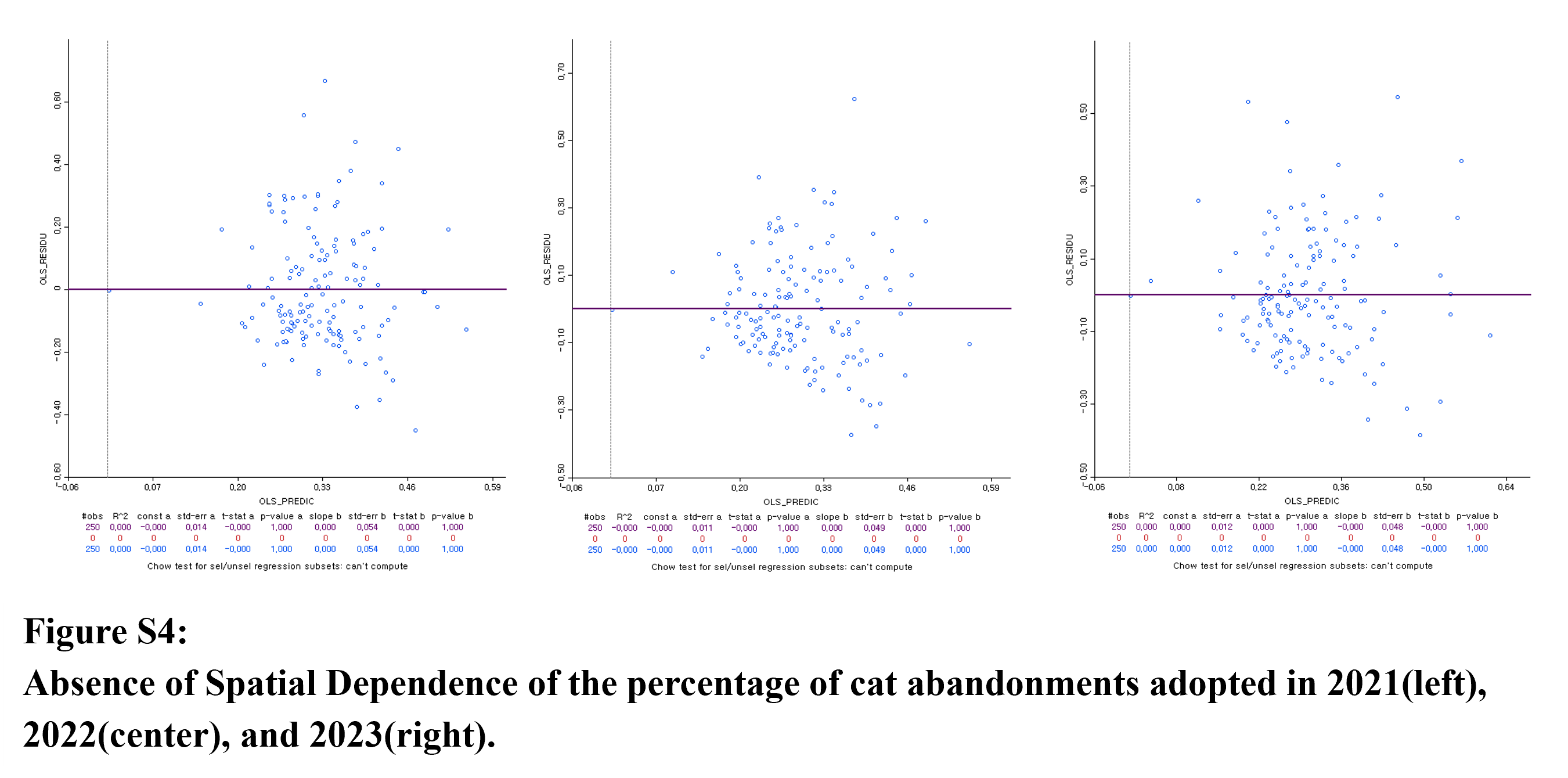

Supplement: Supplemental Information 14 [file peerj-14-21339-s014.png]
