# Supplementary material for: Cat abandonment and adoption associated with socioeconomic, veterinary, and trap–neuter–return factors in the Republic of Korea
Source: PeerJ. 2026 Jun 3;14:e21339. doi: 10.7717/peerj.21339 (PMC13242195; doi:10.7717/peerj.21339)
Supplement: Supplemental Information 15 [file peerj-14-21339-s015.pdf]

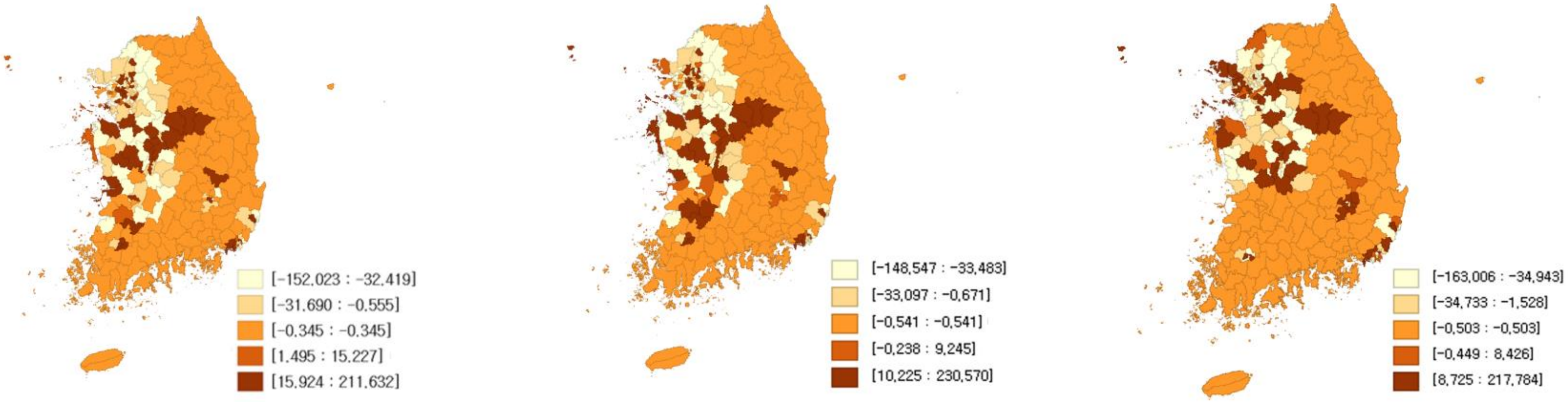

Figure S5:

Absence of Spatial Dependence of the number of cat abandonments per 100,000 residents in 2021(left), 2022(center), and 2023(right).
